# Hepatic and portal vein Dopplers in the clinical management of patients with right-sided heart failure: two case reports

**DOI:** 10.1186/s13089-019-0146-3

**Published:** 2019-11-12

**Authors:** Matthew Jefkins, Barry Chan

**Affiliations:** Division of General Internal Medicine, Department of Medicine, Queen’s University, Kingston, Ontario K7L 3N6 Canada

**Keywords:** Congestive heart failure, Point of care ultrasound, Portal vein, Doppler

## Abstract

**Background:**

Patients with right heart failure pose significant volume management challenges for hemodynamic optimization. We present two cases in which point of care ultrasound (POCUS) of the hepatic and portal veins contributed to the venous hypertension assessment and decongestive strategy for patients with right-sided heart failure.

**Case presentation:**

Patient A was 91 years old with known pulmonary hypertension and right ventricular systolic dysfunction who presented in septic shock requiring vasopressor support. Hepatic and portal vein Dopplers were consistent with right heart failure and significant venous congestion, therefore, diuresis was initiated which resulted in portal flow normalization, renal recovery, and cessation of vasopressor support. Patient B was 82 years old with severe idiopathic pulmonary fibrosis on home oxygen who presented in decompensated right heart failure. Despite aggressive diuresis, a negative fluid balance was not achieved. The patient continued to deteriorate and prior to their death portal vein, Doppler showed significant flow reversal.

**Conclusion:**

Hepatic and portal vein Doppler ultrasounds are venous hypertension assessment tools that can be readily used at the bedside by clinicians trained in POCUS that may contribute holistically to the hemodynamic profiling for patients with right heart failure and direct therapeutic interventions.

## Introduction

Congestive heart failure is a complex condition with effects on other organ systems including liver and kidneys. Patients with right heart failure and concurrent illness such as sepsis pose significant challenges in managing volume status for hemodynamic optimization. Point of care ultrasound (POCUS) is a non-invasive, readily available tool with increasing numbers of clinical applications to assess and help guide management of patients.

In hepatic vein ultrasonography, the normal hepatic waveform is triphasic with 4 components including a retrograde A wave (atrial systole), anterograde S wave (ventricular systole), transitional V wave (atrial overfilling transition wave), and an anterograde D wave (ventricular diastole) [1]. Normally, the magnitude of the S wave is larger than the D wave because the anterograde venous return velocity from the liver to the heart during ventricular systole is higher than the velocity during passive ventricular filling [1]. However, in right heart failure, the hepatic waveform exhibits a S to D wave ratio reversal due to the relatively greater anterograde blood flow during diastole than systole. In addition, tricuspid regurgitation, often coexisting with significant right heart failure, can result in a retrograde S wave [1].

In patients with significant right heart failure, portal vein Doppler demonstrates increased portal vein pulsatility due to an interplay between elevated right atrial pressure and tricuspid regurgitation wave transmission [2]. Conversely, in healthy patients, the portal vein normally shows continuous flow with minimal phasic variation throughout the cardiac cycle [3]. Portal vein pulsatility correlates with New York Heart Association classification, where class III and IV heart failure patients tend to demonstrate increased pulsatility or, at the extreme, flow reversal [2, 4].

The features and characteristics of portal vein Doppler ultrasonography in patients with heart failure have been described in the literature [2, 4, 5], and there have been case reports of clinical application in post-cardiac surgery patients [6] and critical care patients [7]. However, this technique is seldom known to most front-line clinicians. The aim of this report is to illustrate the clinical utility of portal vein Doppler in assessing venous hypertension, guiding the volume management, and the evolution of the waveforms through two patients with right-sided heart failure.

This study was approved by the Queen’s University Health Sciences and Affiliated Teaching Hospitals Research Ethics Board.

## Case presentation

Patient A who was 91 years old with moderate pulmonary hypertension and moderate right ventricular (RV) dysfunction was brought to the hospital for delirium who was found to have acute chronic kidney injury with a creatinine of 262 μmol/L (baseline 120 μmol/L) and in septic shock due to Staphylococcus *epidermidis* bacteremia of unknown source. The patient was initially aggressively volume expanded for hemodynamic resuscitation with no improvement in their mean arterial pressure (MAP), therefore, vasopressor support was started. Norepinephrine was started at 0.05 mcg/kg/min and titrated to a maximum of 0.13 mcg/kg/min to maintain a MAP of 65 mmHg.

**Fig. 1:**
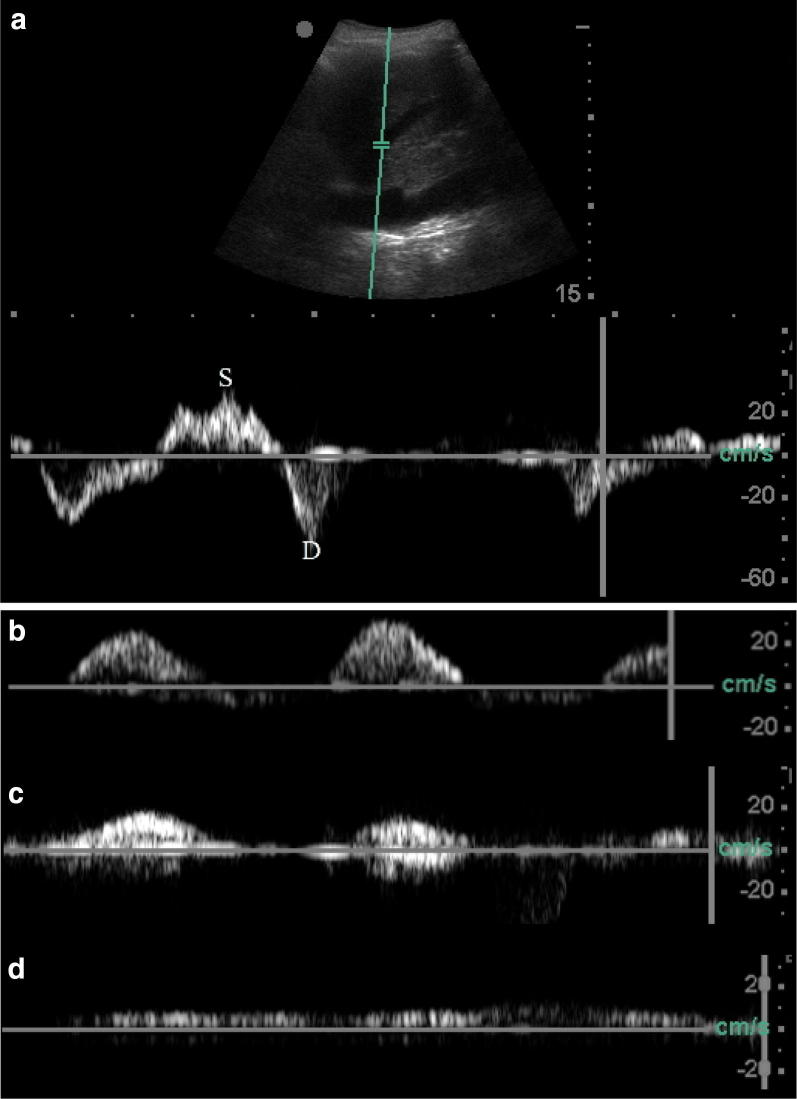
**a** Doppler of the middle hepatic vein on Day 1 demonstrated S wave retrograde flow suggestive of tricuspid regurgitation and/or right heart failure. **b** Doppler of the right portal vein on Day 1 demonstrated pulsatile flow with intermittent flow cessation suggestive of venous congestion. **c** A net negative fluid balance of 1.9 L was achieved which resulted in on Day 2. **d** Then a net negative fluid balance of 4.9 L was achieved which resulted in the normalization to a continuous and anterograde phasic portal flow on Day 3

Given the patient’s predisposition to right heart failure, POCUS was deployed to assess for venous congestion on Day 1. Hepatic vein Doppler revealed D > S wave which suggested tricuspid regurgitation and/or right heart failure (Fig. 1a). Interrogation of the right portal vein, imaged longitudinally from the right mid-axillary line, revealed a pulsatile waveform characterized by intermittent anterograde and cessation of flow suggestive of significant venous congestion (Fig. 1b). Hence, to optimize end-organ perfusion pressure, volume expansion was terminated, and diuresis was pursued. Furosemide 80 mg intravenously once daily was administered on Days 1 and 2 which resulted in net negative fluid balance of 1.9 L and 4.9 L, respectively. Daily reassessment of portal flow demonstrated attenuation of pulsatile flow velocity by Day 2 (Fig. 1c), then a continuous and anterograde phasic flow was achieved by Day 3 (Fig. 1d). Of note, the portal flow velocity on Day 3 was reduced to approximately 10 cm/s (Fig. 1d). This was unlikely secondary to a technical factor as the angle of insonation of the pulse wave Doppler was virtually paralleled with the right portal vein. The cause was most likely secondary to portal hypertension from right heart failure given the clinical context.

The patient was off norepinephrine by Day 2; and signs of renal recovery began on Day 3 with a creatinine of 196 and trending down. The patient was discharged home on post-admission Day 11 with their creatinine recovered to 123 mmol/L.

**Fig. 2:**
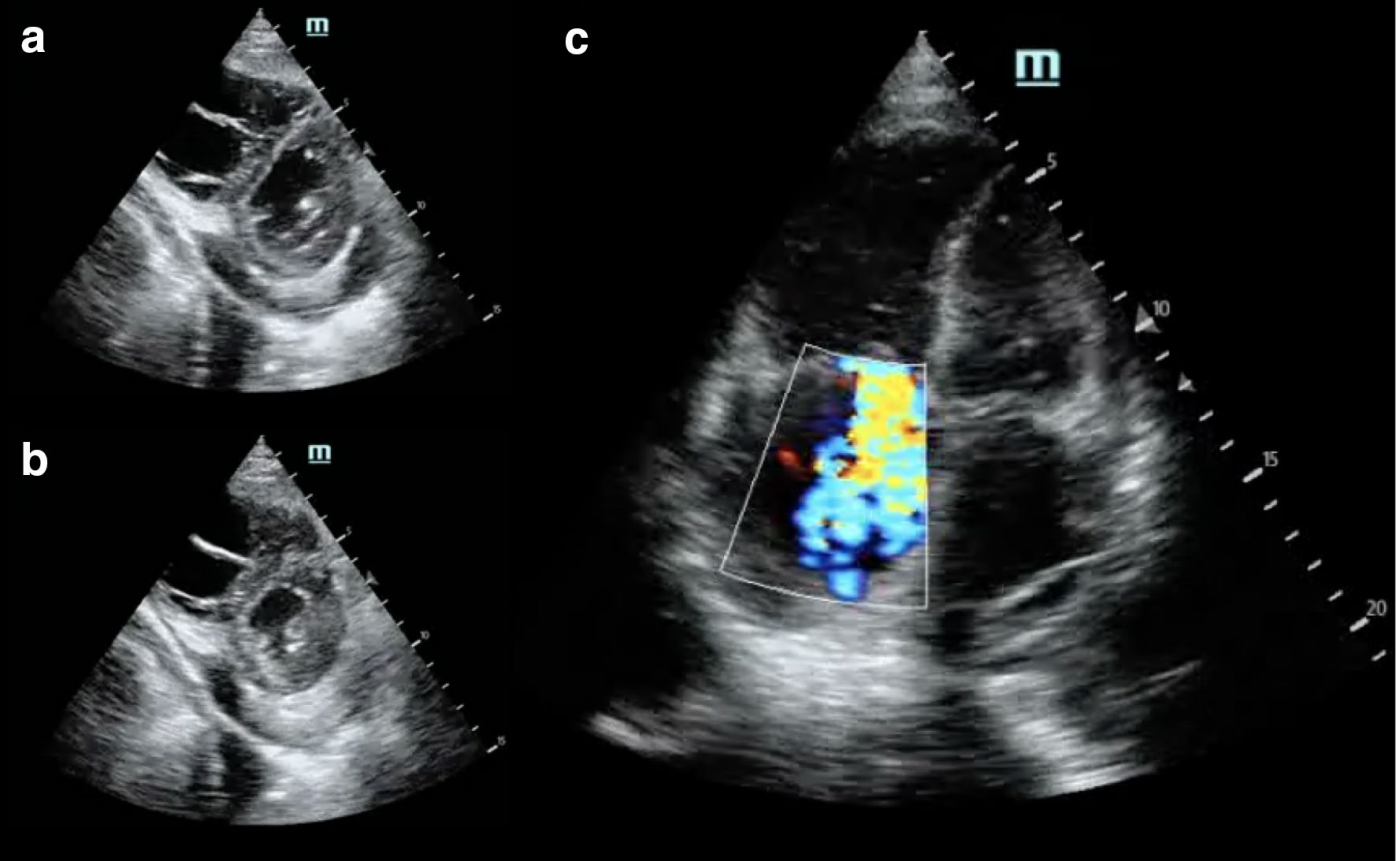
Cardiac POCUS on Day 1, the parasternal short view demonstrated flattening of the interventricular septum only during diastole (**a**) and normalization during systole (**b**). The apical 4 view right ventricle was dilated with evidence of a tricuspid regurgitation jet (**c**)

**Fig. 3:**
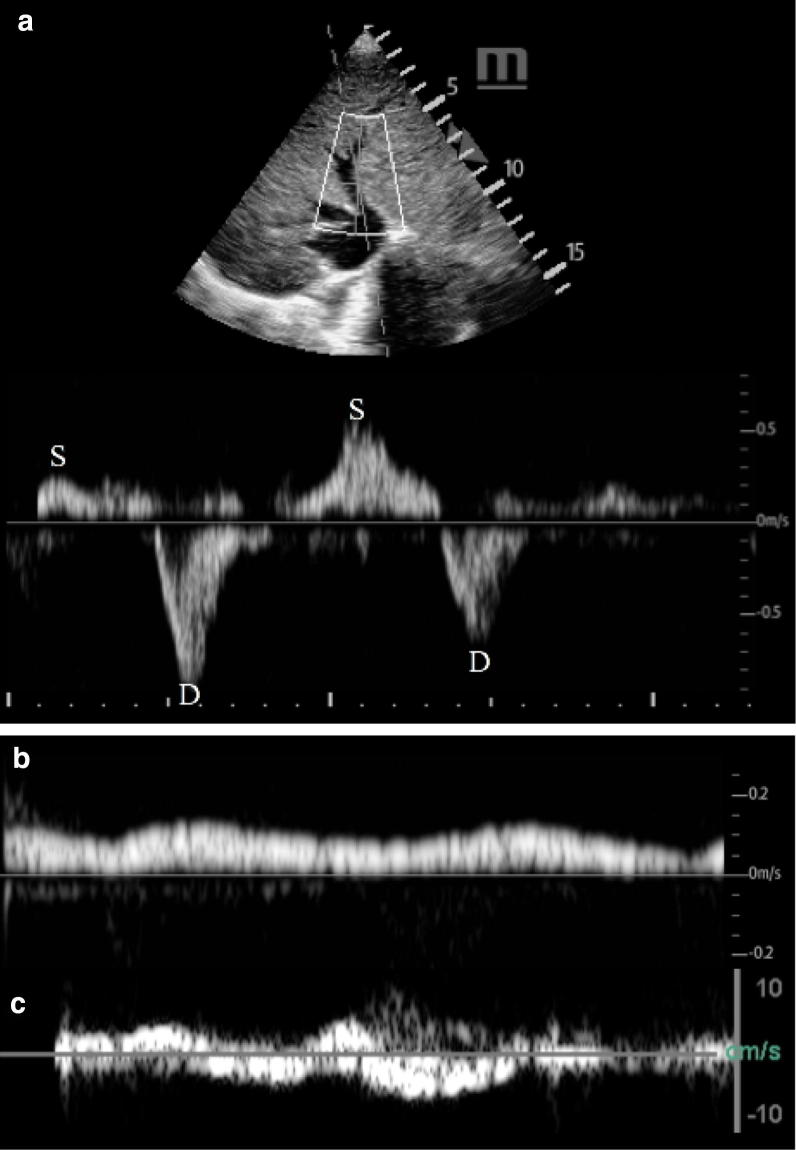
Middle hepatic vein Doppler on Day 1 demonstrated S wave reversal suggestive of tricuspid regurgitation and/or right heart failure (**a**). Right portal vein Doppler on Day 1 demonstrating reduced portal venous flow suggestive of venous congestion (**b**). Right portal vein Doppler on Day 5 demonstrating portal venous flow reversal suggestive of severe heart failure and venous congestion

Patient B was 82 years old with severe idiopathic pulmonary fibrosis (IPF) on 2 L/min home oxygen who was transferred to the hospital for being found hypoxic during a follow-up assessment. Of note, the patient noted increasing peripheral edema up to the proximal thighs that developed over the course of 2 months. CT chest demonstrated features indicative of an IPF flare and bilateral pleural effusions. On Day 1, cardiac POCUS (Fig. 2) revealed a small pericardial effusion, normal LV systolic function, dilated RV, reduced RV systolic function, severe tricuspid regurgitation, and interventricular septal flattening was observed only during diastole. The hepatic vein waveform demonstrated S wave retrograde flow (Fig. 3a) and a continuous yet phasic anterograde portal flow at a lower velocity of about 10 cm/s (Fig. 3b). Given these sonographic findings, right ventricular volume overload and portal venous hypertension were evident; therefore, a decongestive strategy was commenced with 40 mg of intravenous furosemide.

On Day 2, pulsatile flow within the portal vein was present which prompted diuresis escalation from furosemide 40 mg intravenously up to 160 mg. However, the patient failed to respond to high-dose furosemide 160 mg with the addition of metolazone 5 mg orally resulting in daily net volume retention with biochemical evidence of progressive acute kidney injury with creatinine of 111 mmol/L from a baseline of 60 mmol/L, congestive hepatopathy, and an increasing lactic acidosis. On Day 5, the patient developed overt shock whereby the portal vein was in retrograde flow for the majority of time (Fig. 3c). The patient died shortly thereafter.

## Discussion

Volume optimization for patients with right heart failure is a ubiquitous challenge. High right-sided filling pressures and tricuspid regurgitation often confound the interpretation of the jugular venous pulse (JVP) rendering it of little clinical utility for volume management [8]. In addition, the JVP is either not readily visible, or there is substantial inter-examiner variability [9]. Increasing peripheral edema and serial weights are probably the most reliable bedside markers for a clinician. Nevertheless, when a history of change in edema cannot be elicited or reliable serial weights is not available, hepatic and portal vein Doppler are alternative evaluation tools that can be readily deployed. In both cases, the right portal vein was imaged with the same technique where the probe was positioned longitudinally at the level of the right mid-axillary line where the liver is situated.

In Case A, due to the patient’s delirium and lack of collateral information, the aforementioned volume markers could not be obtained. Volume expansion was initiated but given the patient’s right heart failure such an intervention can instead diminish end-organ perfusion due to reduction in cardiac output and venous hypertension. Hepatic and portal vein Dopplers were deployed to resolve this conundrum. The S wave is influenced by the state of compliance of the right atrium during systole and the presence of tricuspid regurgitation. Hence, the abnormal S wave does suggest abnormal right atrial compliance most commonly secondary to right heart dysfunction. The portal vein flow profile revealed a pulsatile flow with intermittent flow cessation suggestive of significantly high pressure downstream. As such, a net negative volume strategy was pursued which resulted in the normalization of the direction of portal flow. This was associated with the subsequent weaning of vasopressor support and renal recovery. The complex mechanisms of cardiointestinal and cardiorenal syndromes may explain these observed associations.

In cardiointestinal syndrome, intestinal hypoperfusion and gut edema are associated with systemic immune activation and increased levels of proinflammatory cytokines as a result of increased gut permeability secondary to gastrointestinal hypoperfusion and gut edema from venous congestion [10]. Hence, decongestive to relief venous hypertension may reduce proinflammatory cytokines and result in vasopressor cessation. In terms of the renal recovery, venous hypertension has been shown to result in increased renal resistance and, subsequently, reduction in renal blood flow. Hence, a net negative volume strategy can improve renal function [11].

Case B illustrated the natural history of decompensated right heart failure through the lens of portal venous flow profile. On presentation, despite having an anterograde and phasic portal venous flow, the reduced velocity indicated evidence of portal hypertension. To distinguish whether it is of cardiac or primary hepatic etiology, clinical context and corroborative investigation are needed [12, 13]. Sonographically, however, the former will be associated severe tricuspid regurgitation and concomitant S to D wave ratio reversal of the hepatic vein whereas the portal hypertension secondary to a primary liver disease should not have these associated findings. In this case, despite aggressive diuresis, daily net positive balance resulted. Eventually, the venous hypertension was so severe such that the portal vein flow reversed which signified severely decompensated right-sided heart failure [4].

Both cases illustrate the utility of hepatic and portal venous Doppler to assess for venous hypertension for patients with right heart failure. Beaubien-Souligny et al. [6] demonstrated an association between portal vein pulsatility and the risk of acute kidney injury as manifested by venous congestion in a prospective cohort study in postoperative cardiac surgery patients. Similar to our cases, they found that portal vein pulsatility was associated with S to D wave ratio reversal of the hepatic vein waveform.

In terms of therapeutic intervention, for our two cases, a decongestive strategy was pursued to alleviate venous congestion to improve end-organ perfusion. Nevertheless, the added sonographic information from both hepatic and portal vein Doppler cannot be interpreted in isolation—they contribute to the assessment of a patient’s hemodynamic profile holistically. Even if the portal flow demonstrates pulsatility or flow reversal, volume removal maybe deleterious should a high preload state is required to maintain cardiac output such as massive pulmonary embolism, or cardiac tamponade. The former requires afterload reduction and the latter necessitates relief from the intrapericardial pressure.

## Conclusion

Hepatic and portal vein Doppler interrogations are venous hypertension assessment adjuncts for patients with right heart failure which may direct therapeutic interventions in conjunction with other hemodynamic parameters.

## Data Availability

All scans obtained are available via the corresponding author.

## Abbreviations

IPF: idiopathic pulmonary fibrosis
JVP: jugular venous pulse
MAP: mean arterial pressure
NYHA: New York Heart Association
POCUS: point of care ultrasound
RV: right ventricle/right ventricular
